# Supplementary material for: Evolution of Plant Na+-P-Type ATPases: From Saline Environments to Land Colonization
Source: Plants (Basel). 2021 Jan 24;10(2):221. doi: 10.3390/plants10020221 (PMC7911474; doi:10.3390/plants10020221)
Supplement: Supplementary file 1 [file plants-10-00221-s001.zip › Supplementary figure 6_site evolution.pdf]

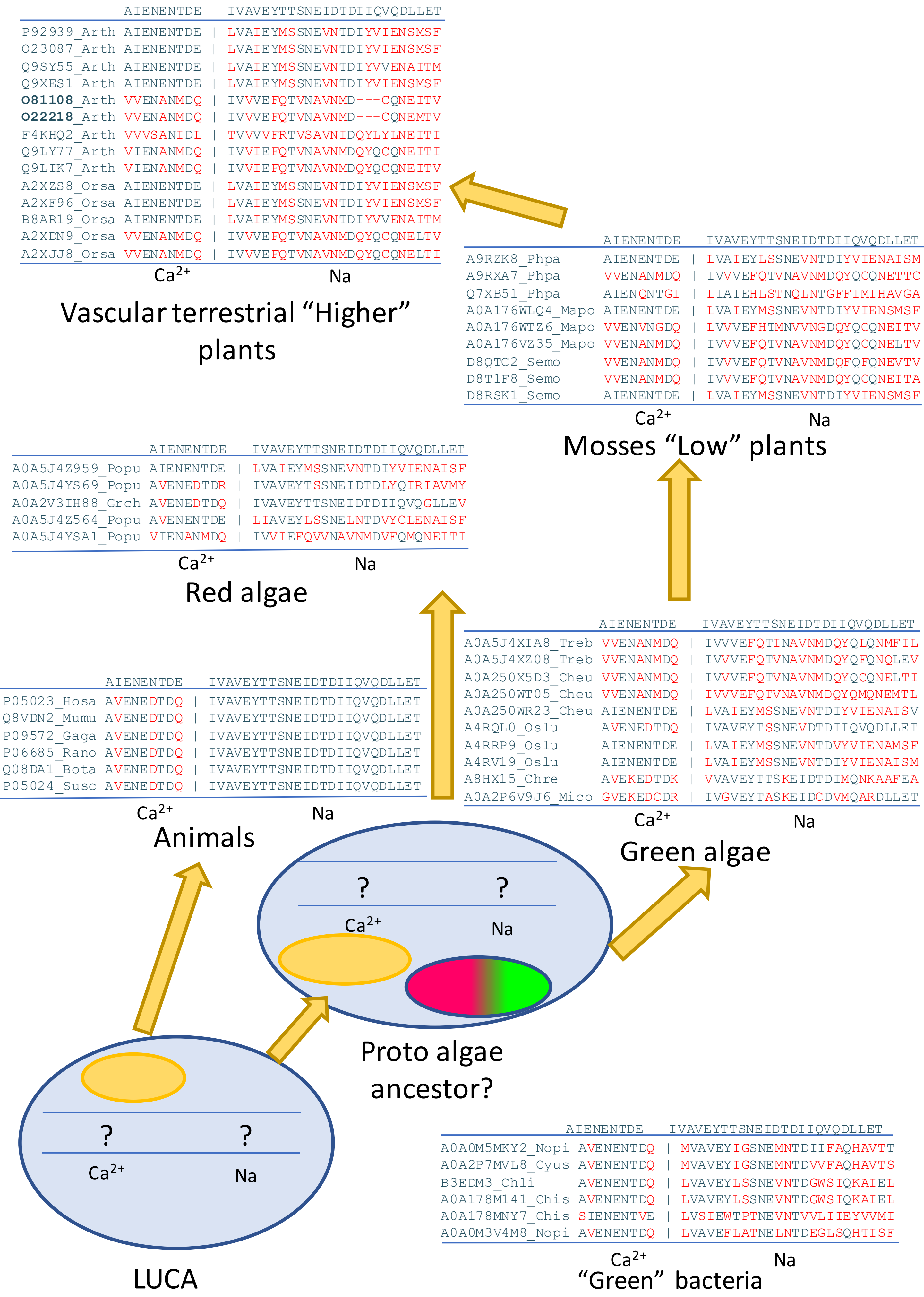

AIENENTDE IVAVEYTTSNEIDTDIIQVQDLLET
P92939_Arth AIENENTDE | LVAIEYMSSNEVNTDIYVIENSMSF
O23087_Arth AIENENTDE | LVAIEYMSSNEVNTDIYVIENSMSF
Q9SY55_Arth AIENENTDE | LVAIEYMSSNEVNTDIYVVENAITM
Q9XES1_Arth AIENENTDE | LVAIEYMSSNEVNTDIYVIENSMSF
O81108_Arth VVENANMDQ | IVVVEFQTVNAVNMD---CQNEITV
O22218_Arth VVENANMDQ | IVVVEFQTVNAVNMD---CQNEMTV
F4KHQ2_Arth VVVSANIDL | TVVVVFRTVSAVNIDQYLYLNEITI
Q9LY77_Arth VIENANMDQ | IVVIEFQTVNAVNMDQYQCQNEITI
Q9LIK7_Arth VIENANMDQ | IVVIEFQTVNAVNMDQYQCQNEITV
A2XZS8_Orsa AIENENTDE | LVAIEYMSSNEVNTDIYVIENSMSF
A2XF96_Orsa AIENENTDE | LVAIEYMSSNEVNTDIYVIENSMSF
B8AR19_Orsa AIENENTDE | LVAIEYMSSNEVNTDIYVVENAITM
A2XDN9_Orsa VVENANMDQ | IVVVEFQTVNAVNMDQYQCQNELTV
A2XJJ8_Orsa VVENANMDQ | IVVVEFQTVNAVNMDQYQCQNELTI
Ca2+
Na
Vascular terrestrial “Higher” plants
AIENENTDE IVAVEYTTSNEIDTDIIQVQDLLET
A9RZK8_Phpa AIENENTDE | LVAIEYLSSNEVNTDIYVIENAISM
A9RXA7_Phpa VVENANMDQ | IVVVEFQTVNAVNMDQYQCQNETTC
Q7XB51_Phpa AIENQNTGI | LIAIEHLSTNQLNTGFFIMIHAVGA
A0A176WLQ4_Mapo AIENENTDE | LVAIEYMSSNEVNTDIYVIENSMSF
A0A176WTZ6_Mapo VVENVNGDQ | LVVVEFHTMNVVNGDQYQCQNEITV
A0A176VZ35_Mapo VVENANMDQ | IVVVEFQTVNAVNMDQYQCQNELTV
D8QTC2_Semo VVENANMDQ | IVVVEFQTVNAVNMDQFQFQNEVTV
D8T1F8_Semo VVENANMDQ | IVVVEFQTVNAVNMDQYQCQNEITA
D8RSK1_Semo AIENENTDE | LVAIEYMSSNEVNTDIYVIENSMSF
Ca2+
Na
Mosses “Low” plants
AIENENTDE IVAVEYTTSNEIDTDIIQVQDLLET
A0A5J4Z959_Popu AIENENTDE | LVAIEYMSSNEVNTDIYVIENAISF
A0A5J4YS69_Popu AVENEDTDR | IVAVEYTSSNEIDTDLYQIRIAVMY
A0A2V3IH88_Grch AVENEDTDQ | IVAVEYTTSNEIDTDIIQVQGLLEV
A0A5J4Z564_Popu AVENENTDE | LIAVEYLSSNELNTDVYCLENAISF
A0A5J4YSA1_Popu VIENANMDQ | IVVIEFQVVNAVNMDVFQMQNEITI
Ca2+
Na
Red algae
AIENENTDE IVAVEYTTSNEIDTDIIQVQDLLET
P05023_Hosa AVENEDTDQ | IVAVEYTTSNEIDTDIIQVQDLLET
Q8VDN2_Mumu AVENEDTDQ | IVAVEYTTSNEIDTDIIQVQDLLET
P09572_Gaga AVENEDTDQ | IVAVEYTTSNEIDTDIIQVQDLLET
P06685_Rano AVENEDTDQ | IVAVEYTTSNEIDTDIIQVQDLLET
Q08DA1_Bota AVENEDTDQ | IVAVEYTTSNEIDTDIIQVQDLLET
P05024_Susc AVENEDTDQ | IVAVEYTTSNEIDTDIIQVQDLLET
Ca2+
Na
Animals
AIENENTDE IVAVEYTTSNEIDTDIIQVQDLLET
A0A5J4XIA8_Treb VVENANMDQ | IVVVEFQTINAVNMDQYQLQNMFIL
A0A5J4XZ08_Treb VVENANMDQ | IVVVEFQTVNAVNMDQYQFQNQLEV
A0A250X5D3_Cheu VVENANMDQ | IVVVEFQTVNAVNMDQYQCQNELTI
A0A250WT05_Cheu VVENANMDQ | IVVVEFQTVNAVNMDQYQMQNEMTL
A0A250WR23_Cheu AIENENTDE | LVAIEYMSSNEVNTDIYVIENAISV
A4RQL0_Oslu AVENEDTDQ | IVAVEYTSSNEVDTDIIQVQDLLET
A4RRP9_Oslu AIENENTDE | LVAIEYMSSNEVNTDVYVIENAMSF
A4RV19_Oslu AIENENTDE | LVAIEYMSSNEVNTDIYVIENAISM
A8HX15_Chre AVEKEDTDK | VVAVEYTTSKEIDTDIMQNKAAFEA
A0A2P6V9J6_Mico GVEKEDCDR | IVGVEYTASKEIDCDVMQARDLLET
Ca2+
Na
Green algae
?
?
Ca2+
Na
Proto algae ancestor?
?
?
Ca2+
Na
LUCA
AIENENTDE IVAVEYTTSNEIDTDIIQVQDLLET
A0A0M5MKY2_Nopi AVENENTDQ | MVAVEYIGSNEMNTDIIFAQHAVTT
A0A2P7MVL8_Cyus AVENENTDQ | MVAVEYIGSNEMNTDVVFAQHAVTS
B3EDM3_Chli AVENENTDQ | LVAVEYLSSNEVNTDGWSIQKAIEL
A0A178M141_Chis AVENENTDQ | LVAVEYLSSNEVNTDGWSIQKAIEL
A0A178MNY7_Chis SIENENTVE | LVSIEWTPTNEVNTVVLIIEYVVMI
A0A0M3V4M8_Nopi AVENENTDQ | LVAVEFLATNELNTDEGLSQHTISF
Ca2+
Na
“Green” bacteria
