# Supplementary material for: Evolution of Plant Na+-P-Type ATPases: From Saline Environments to Land Colonization
Source: Plants (Basel). 2021 Jan 24;10(2):221. doi: 10.3390/plants10020221 (PMC7911474; doi:10.3390/plants10020221)
Supplement: Supplementary file 1 [file plants-10-00221-s001.zip › Supplementary materials+legends-1N.pdf]

Supplementary Materials

Evolution of Plant Na^+-P-Type ATPases: From Saline Environments to Land Colonization

Siarhei A. Dabravolski [1] and Stanislav V. Isayenkov [2,3,*]

[1] Department of Clinical Diagnostics, Vitebsk State Academy of Veterinary Medicine [UO VGAVM], Vitebsk, 21002, Belarus; sergedobrowolski@gmail.com
[2] International Research Centre for Environmental Membrane Biology, Foshan University, Foshan, China
[3] Department of Plant Food Products and Biofortification, Institute of Food Biotechnology and Genomics NAS of Ukraine, Kyiv, Ukraine
* Correspondence: author: stan.isayenkov@gmail.com

Figure captions

Supplementary Table S1. 1. Domain-based classification of the P-ATPases from species used in this study. Known Na^+/K^+-transporting P-ATPases are marked with green. Unusual metal-binding domain (PF12156) is marked with yellow. 2. List of species used in this study. Taxonomy was simplified. 3. List of Na^+/K^+-transporting ATPases from green and red Algae and Animalia used to extract β-like subunit insertions (algae) and compare to the β subunit from Animalia. 4. List of Bacteria species with β subunit and algae with β-like subunit insertions.

Supplementary Figure S1. **(a)** Comparison of Ca^{2+} (depicted in yellow circles) binding sites from known sodium/potassium-transporting P-ATPases (identified by [51,56]). AtACA4 and AtACA2 proteins, known to be involved in NaCl tolerance [71,72], highlighted in bold. **(b)** Comparison of Na+ binding sites from known sodium/potassium-transporting P-ATPases identified by [51] (depicted in magenta circles) and [57] (depicted in green squares). AtACA4 and AtACA2 proteins, known to be involved in NaCl tolerance [72,73], highlighted in bold. **(c)** The first C-terminal insertion into the green (*Ostreococcus lucimarinus*, *Micractinium conductrix*, and *Chlamydomonas reinhardtii*) and red (*Porphyridium purpureum* and *Gracilariopsis chorda*) algae's α-subunit with a high similarity to the animal's β-subunit. **(d)** Conserved catalytic D site, identified in α-subunit, depicted in magenta. AtACA4 and AtACA2 proteins known to be involved in NaCl tolerance [72,73], highlighted in bold. **(e)** Amino acids of the α-subunit important for the interaction with γ-subunit (identified in *Sus scrofa* (4HQJ) [57], depicted with yellow dots). AtACA4 and AtACA2 proteins, known to be involved in NaCl tolerance [72,73], are highlighted in bold.

Supplementary Figure S2. Structural alignment of the β-subunits from human (P05026) (magenta), *Anthurium amnicola* (A0A1D1Y1U2) (green) and *Gammaproteobacteria bacterium 2W06* (PYZ99283.1) (cyan). Alignment scores: (A) human vs. bacteria 164.32, RMSD 0.70; (B) human vs. plant 182.51, RMSD 0.67; (C) bacteria vs. plant 305.99, RMSD 0.98. iPBA webserver was used for the pdb structures alignment (https://www.dsimb.inserm.fr/dsimb_tools/ipba/index.php).

Supplementary Figure S3. The second C-terminal insertion into the green (*Ostreococcus lucimarinus*, *Micractinium conductrix* and *Chlamydomonas reinhardtii*) and red (*Porphyridium purpureum* and *Gracilariopsis chorda*) algae α-subunit with high similarity to the animal's β-subunit.

Supplementary Figure S4. Amino acids of the α-subunit important for the interaction with β-subunit (identified in *Sus scrofa* (4HQJ) [57]), depicted with blue dots. AtACA4 and AtACA2 proteins, known to be involved in NaCl tolerance [72,73], highlighted in bold.

Supplementary Figure S5. Alignment of the β-subunit-like insertions of the algae (highlighted in bold) with β-subunits from animals, *Anthurium amnicola* (A0A1D1Y1U2), and *Gammaproteobacteria bacterium 2W06* (PYZ99283.1). Amino acids, important for the interaction with β-subunit (depicted with blue dots) and γ-subunit (depicted with orange dots) identified in *Sus scrofa* (4HQJ) [57].

Supplementary Figure S6. Sites evolution. Evolution of the Ca^{2+} and Na^+ binding sites throughout the simplified tree of life. LUCA (last unicellular common ancestor) represents an ancient cell-structure before the split on the "plant" and "animal" lines. The fungi line is not presented. The evolution of the plant started from the incorporation of the cyanobacteria to the ancestor (proto algae), giving rise to the red and green algae lineages. Further evolution line includes "low" plants (mosses) and flowering plants.

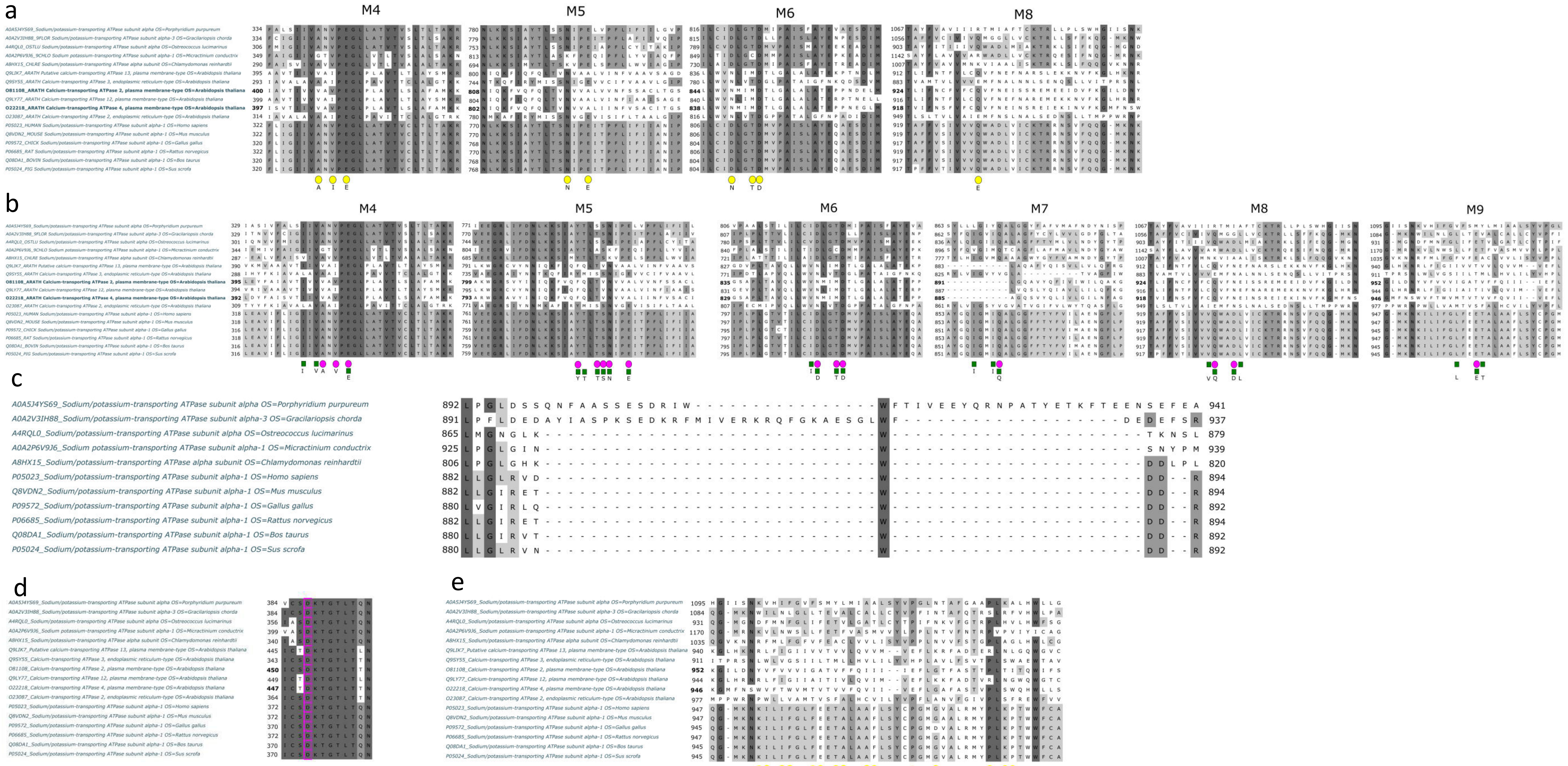
a
M4
M5
M6
M8
A I E
N E
N T D
E
b
M4
M5
M6
M7
M8
M9
I V A V E
Y T T S N E
I D T D
I Q
V Q D L
L E T
c
d
e
K I I F F E T A A F P G P K P

Figure S1

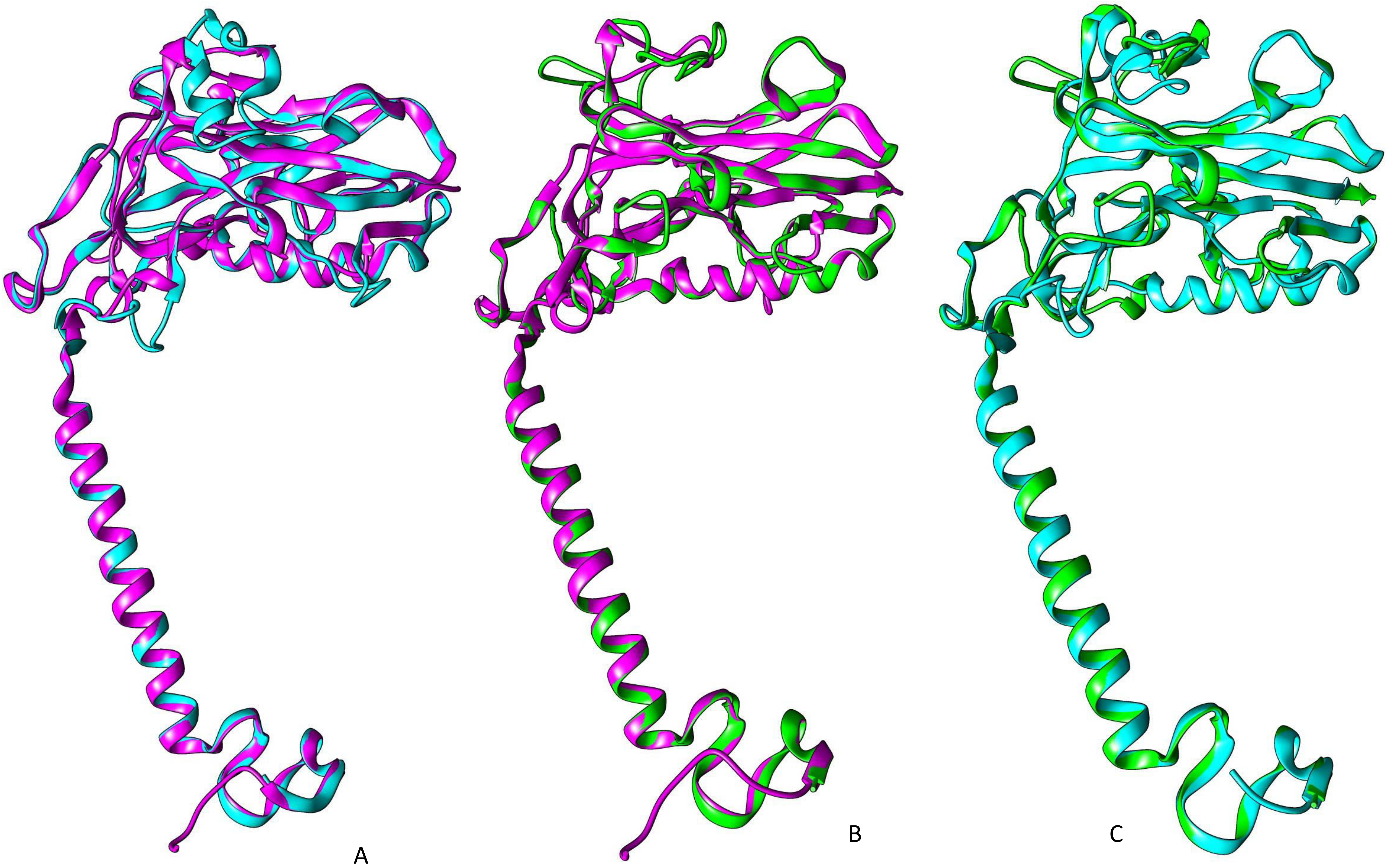

Figure S2

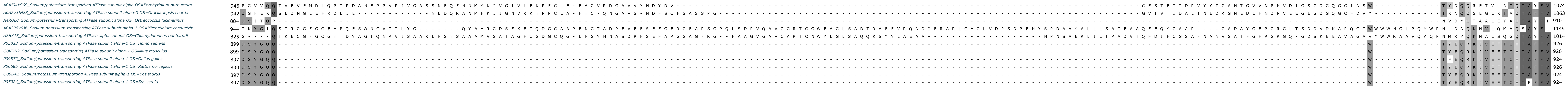
A0A5J4YS69_Sodium/potassium-transporting ATPase subunit alpha OS=Porphyridium purpureum
A0A2V3IH88_Sodium/potassium-transporting ATPase subunit alpha-3 OS=Gracilariopsis chorda
A4RQL0_Sodium/potassium-transporting ATPase subunit alpha OS=Ostreococcus lucimarinus
A0A2P6V9J6_Sodium potassium-transporting ATPase subunit alpha-1 OS=Micractinium conductrix
A8HX15_Sodium/potassium-transporting ATPase alpha subunit OS=Chlamydomonas reinhardtii
P05023_Sodium/potassium-transporting ATPase subunit alpha-1 OS=Homo sapiens
Q8VDN2_Sodium/potassium-transporting ATPase subunit alpha-1 OS=Mus musculus
P09572_Sodium/potassium-transporting ATPase subunit alpha-1 OS=Gallus gallus
P06685_Sodium/potassium-transporting ATPase subunit alpha-1 OS=Rattus norvegicus
Q08DA1_Sodium/potassium-transporting ATPase subunit alpha-1 OS=Bos taurus
P05024_Sodium/potassium-transporting ATPase subunit alpha-1 OS=Sus scrofa

Figure S3

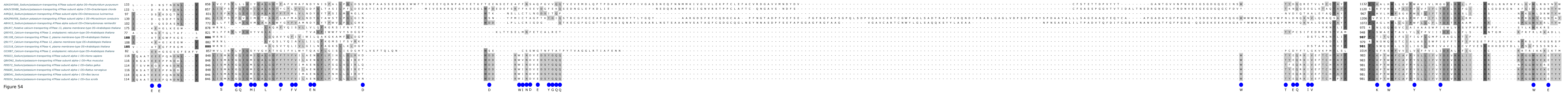

Figure S4

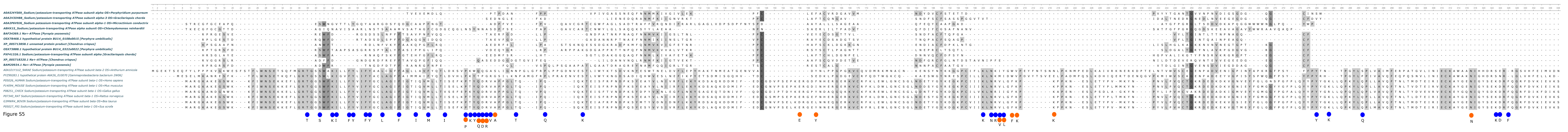

Figure S5

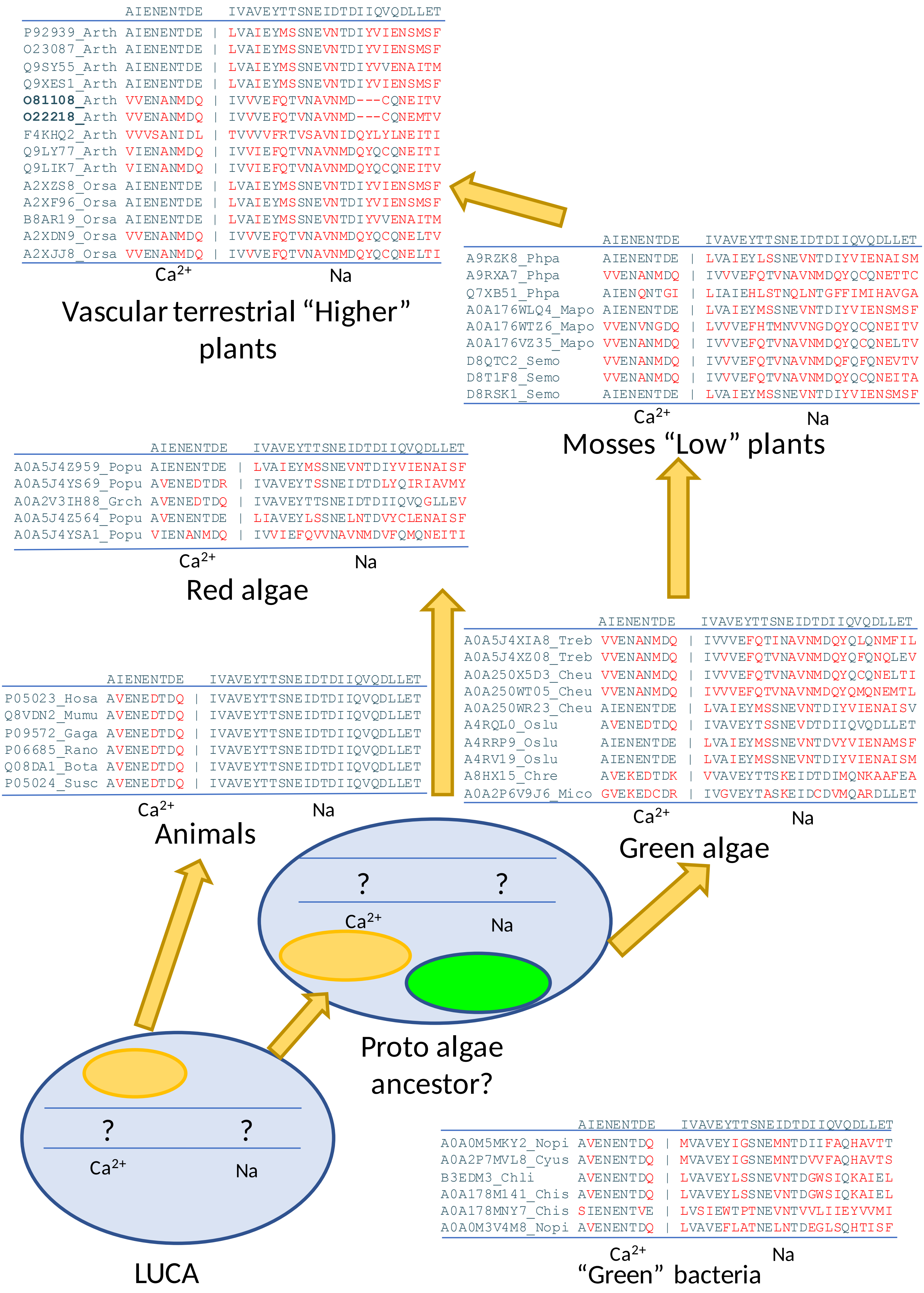
AIENENTDE IVAVEYTTSNEIDTDIIQVQDLLET
P92939_Arth AIENENTDE | LVAIEYMSSNEVNTDIYVIENSMSF
O23087_Arth AIENENTDE | LVAIEYMSSNEVNTDIYVIENSMSF
Q9SY55_Arth AIENENTDE | LVAIEYMSSNEVNTDIYVVENAITM
Q9XES1_Arth AIENENTDE | LVAIEYMSSNEVNTDIYVIENSMSF
O81108_Arth VVENANMDQ | IVVVEFQTVNAVNMD---CQNEITV
O22218_Arth VVENANMDQ | IVVVEFQTVNAVNMD---CQNEMTV
F4KHQ2_Arth VVVSANIDL | TVVVVFRTVSAVNIDQYLYLNEITI
Q9LY77_Arth VIENANMDQ | IVVIEFQTVNAVNMDQYQCQNEITI
Q9LIK7_Arth VIENANMDQ | IVVIEFQTVNAVNMDQYQCQNEITV
A2XZS8_Orsa AIENENTDE | LVAIEYMSSNEVNTDIYVIENSMSF
A2XF96_Orsa AIENENTDE | LVAIEYMSSNEVNTDIYVIENSMSF
B8AR19_Orsa AIENENTDE | LVAIEYMSSNEVNTDIYVVENAITM
A2XDN9_Orsa VVENANMDQ | IVVVEFQTVNAVNMDQYQCQNELTV
A2XJJ8_Orsa VVENANMDQ | IVVVEFQTVNAVNMDQYQCQNELTI
Ca2+ Na
Vascular terrestrial "Higher" plants
AIENENTDE IVAVEYTTSNEIDTDIIQVQDLLET
A9RZK8_Phpa AIENENTDE | LVAIEYLSSNEVNTDIYVIENAISM
A9RXA7_Phpa VVENANMDQ | IVVVEFQTVNAVNMDQYQCQNETTC
Q7XB51_Phpa AIENQNTGI | LIAIEHLSTNQLNTGFFIMIHAVGA
A0A176WLQ4_Mapo AIENENTDE | LVAIEYMSSNEVNTDIYVIENSMSF
A0A176WTZ6_Mapo VVENVNGDQ | LVVVEFHTMNVVNGDQYQCQNEITV
A0A176VZ35_Mapo VVENANMDQ | IVVVEFQTVNAVNMDQYQCQNELTV
D8QTC2_Semo VVENANMDQ | IVVVEFQTVNAVNMDQFQFQNEVTV
D8T1F8_Semo VVENANMDQ | IVVVEFQTVNAVNMDQYQCQNEITA
D8RSK1_Semo AIENENTDE | LVAIEYMSSNEVNTDIYVIENSMSF
Ca2+ Na
Mosses "Low" plants
AIENENTDE IVAVEYTTSNEIDTDIIQVQDLLET
A0A5J4Z959_Popu AIENENTDE | LVAIEYMSSNEVNTDIYVIENAISF
A0A5J4YS69_Popu AVENEDTDR | IVAVEYTSSNEIDTDLYQIRIAVMY
A0A2V3IH88_Grch AVENEDTDQ | IVAVEYTTSNEIDTDIIQVQGLLEV
A0A5J4Z564_Popu AVENENTDE | LIAVEYLSSNELNTDVYCLENAISF
A0A5J4YSA1_Popu VIENANMDQ | IVVIEFQVVNAVNMDVFQMQNEITI
Ca2+ Na
Red algae
AIENENTDE IVAVEYTTSNEIDTDIIQVQDLLET
P05023_Hosa AVENEDTDQ | IVAVEYTTSNEIDTDIIQVQDLLET
Q8VDN2_Mumu AVENEDTDQ | IVAVEYTTSNEIDTDIIQVQDLLET
P09572_Gaga AVENEDTDQ | IVAVEYTTSNEIDTDIIQVQDLLET
P06685_Rano AVENEDTDQ | IVAVEYTTSNEIDTDIIQVQDLLET
Q08DA1_Bota AVENEDTDQ | IVAVEYTTSNEIDTDIIQVQDLLET
P05024_Susc AVENEDTDQ | IVAVEYTTSNEIDTDIIQVQDLLET
Ca2+ Na
Animals
AIENENTDE IVAVEYTTSNEIDTDIIQVQDLLET
A0A5J4XIA8_Treb VVENANMDQ | IVVVEFQTINAVNMDQYQLQNMFIL
A0A5J4XZ08_Treb VVENANMDQ | IVVVEFQTVNAVNMDQYQFQNQLEV
A0A250X5D3_Cheu VVENANMDQ | IVVVEFQTVNAVNMDQYQCQNELTI
A0A250WT05_Cheu VVENANMDQ | IVVVEFQTVNAVNMDQYQMQNEMTL
A0A250WR23_Cheu AIENENTDE | LVAIEYMSSNEVNTDIYVIENAISV
A4RQL0_Oslu AVENEDTDQ | IVAVEYTSSNEVDTDIIQVQDLLET
A4RRP9_Oslu AIENENTDE | LVAIEYMSSNEVNTDVYVIENAMSF
A4RV19_Oslu AIENENTDE | LVAIEYMSSNEVNTDIYVIENAISM
A8HX15_Chre AVEKEDTDK | VVAVEYTTSKEIDTDIMQNKAAFEA
A0A2P6V9J6_Mico GVEKEDCDR | IVGVEYTASKEIDCDVMQARDLLET
Ca2+ Na
Green algae
? ?
Ca2+ Na
Proto algae ancestor?
? ?
Ca2+ Na
LUCA
AIENENTDE IVAVEYTTSNEIDTDIIQVQDLLET
A0A0M5MKY2_Nopi AVENENTDQ | MVAVEYIGSNEMNTDIIFAQHAVTT
A0A2P7MVL8_Cyus AVENENTDQ | MVAVEYIGSNEMNTDVVFAQHAVTS
B3EDM3_Chli AVENENTDQ | LVAVEYLSSNEVNTDGWSIQKAIEL
A0A178M141_Chis AVENENTDQ | LVAVEYLSSNEVNTDGWSIQKAIEL
A0A178MNY7_Chis SIENENTVE | LVSIEWTPTNEVNTVVLIIEYVVMI
A0A0M3V4M8_Nopi AVENENTDQ | LVAVEFLATNELNTDEGLSQHTISF
Ca2+ Na
"Green" bacteria

Figure S6
