# Supplementary figures and images for: Evolution of Plant Na+-P-Type ATPases: From Saline Environments to Land Colonization
Source: Plants (Basel). 2021 Jan 24;10(2):221. doi: 10.3390/plants10020221 (PMC7911474; doi:10.3390/plants10020221)

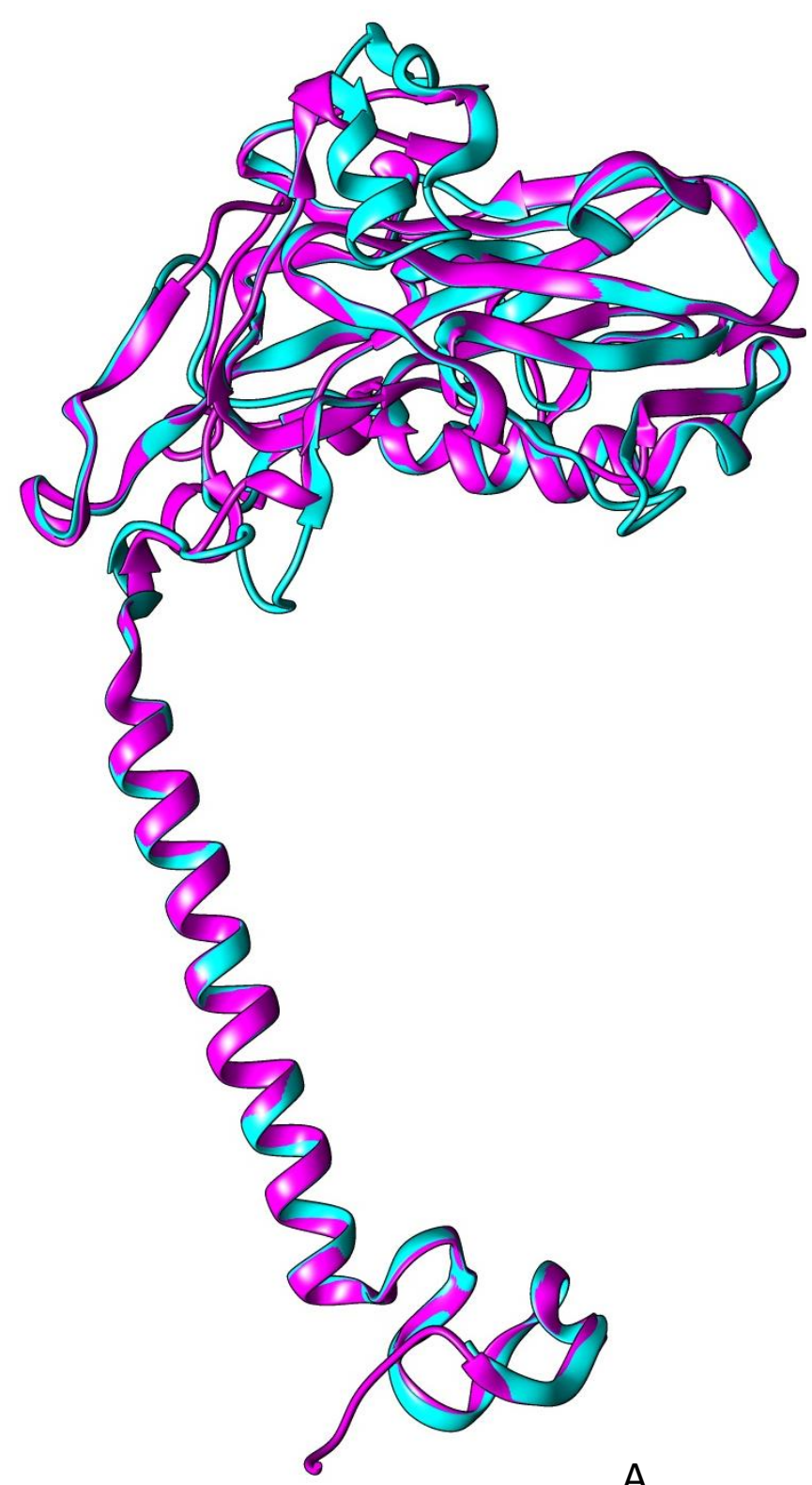

A

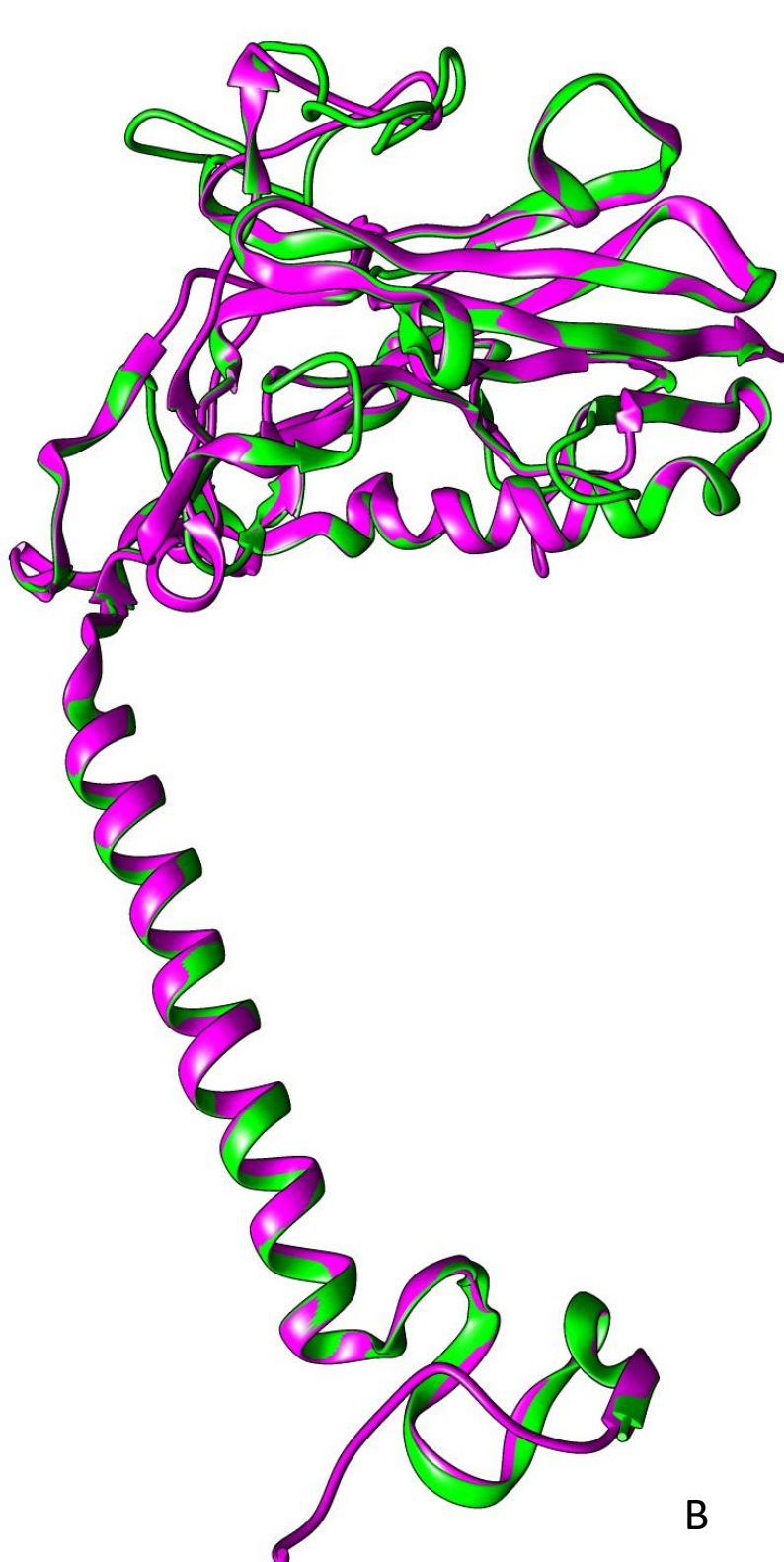

B

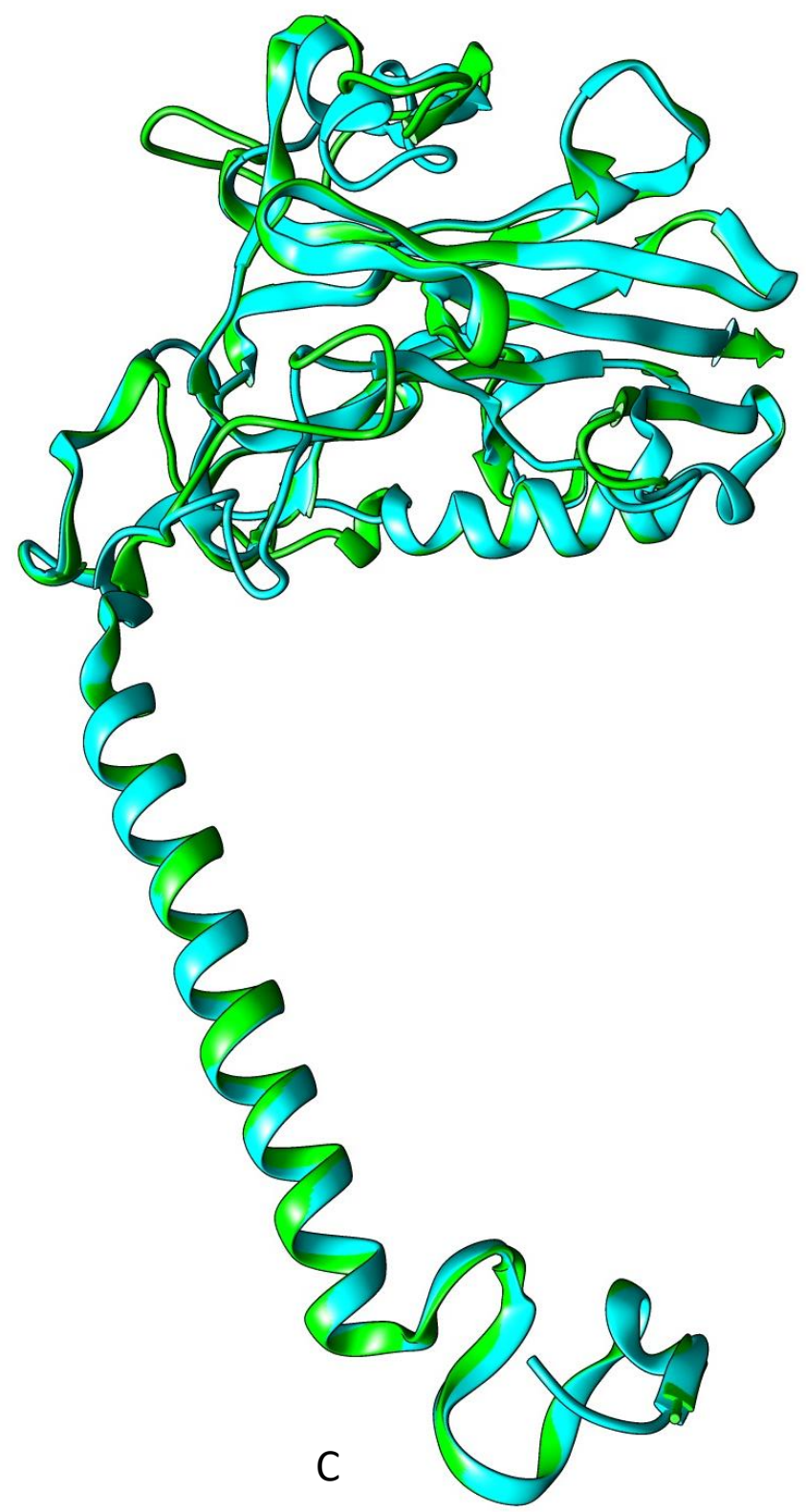

C

Supplement: Supplementary file 1 [file plants-10-00221-s001.zip › Supplementary figure 2_alignment-3D.pdf]

## Slide 1
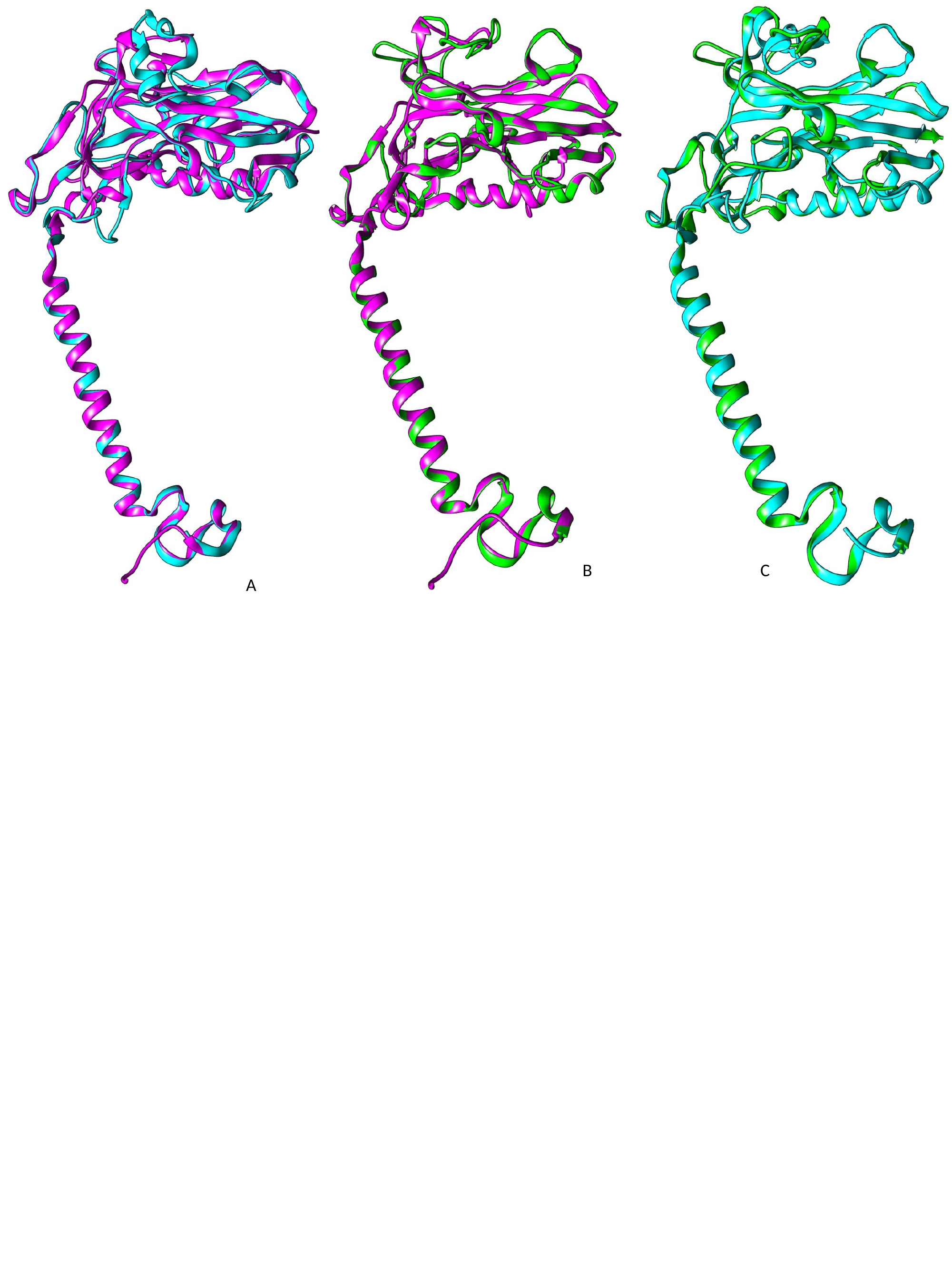

C
B
A

Supplement: Supplementary file 1 [file plants-10-00221-s001.zip › Supplementary figure 2_alignment-3D.pptx]

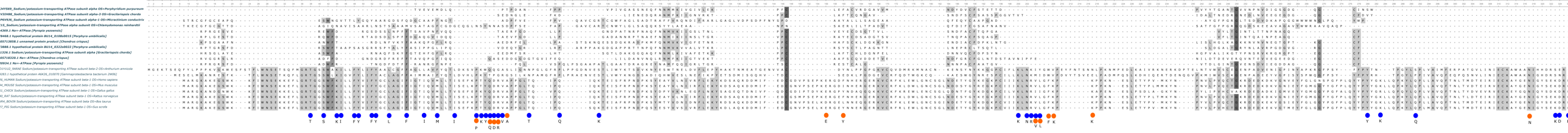

Supplement: Supplementary file 1 [file plants-10-00221-s001.zip › Supplementary figure 5_Inter-b.pdf]

a

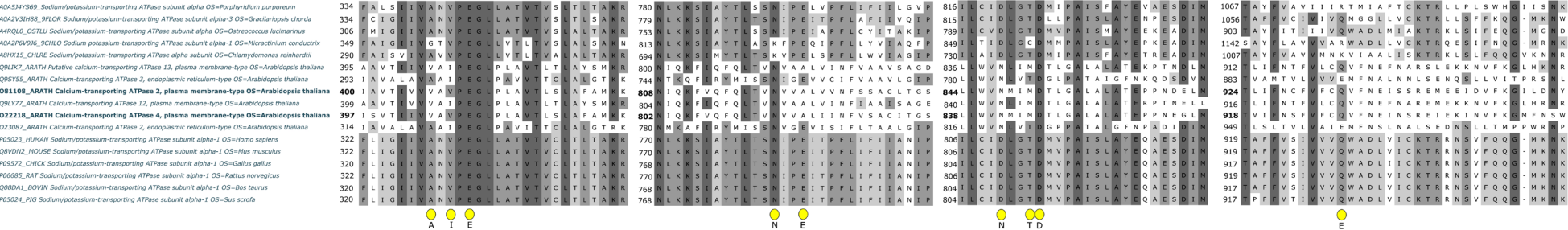

b

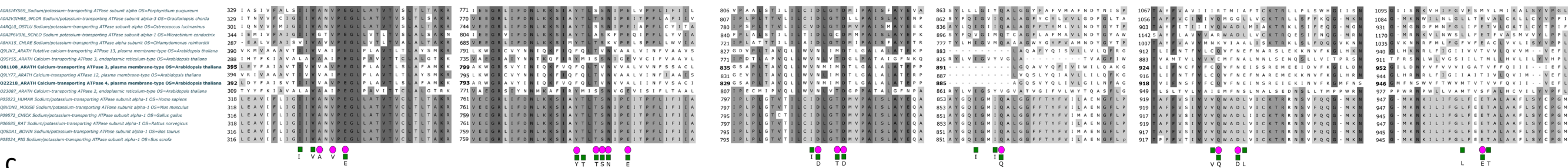

c

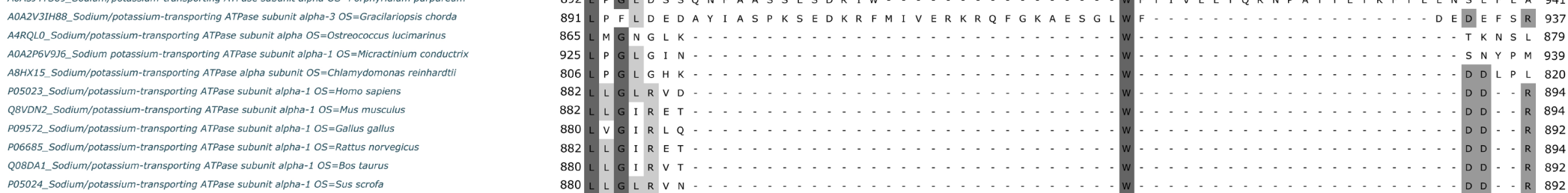

d

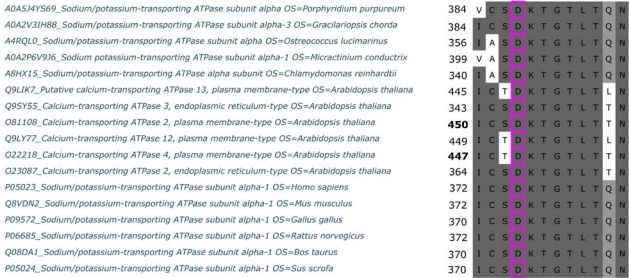

e

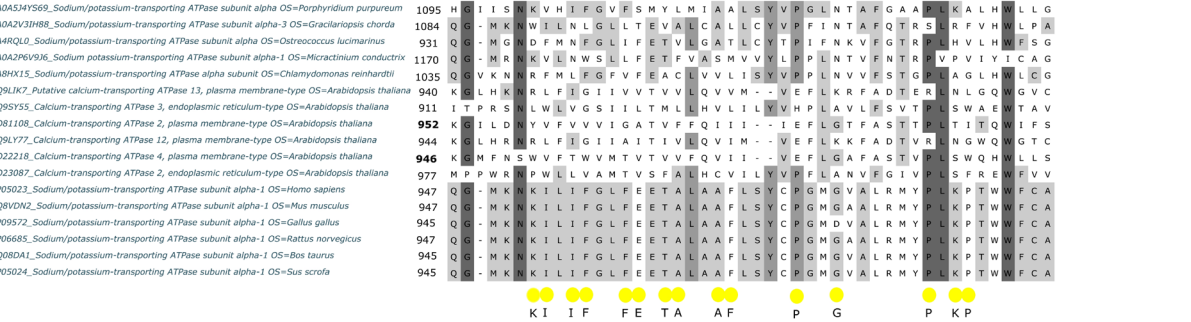

Supplement: Supplementary file 1 [file plants-10-00221-s001.zip › Supplementary figure 1_R2.pdf]

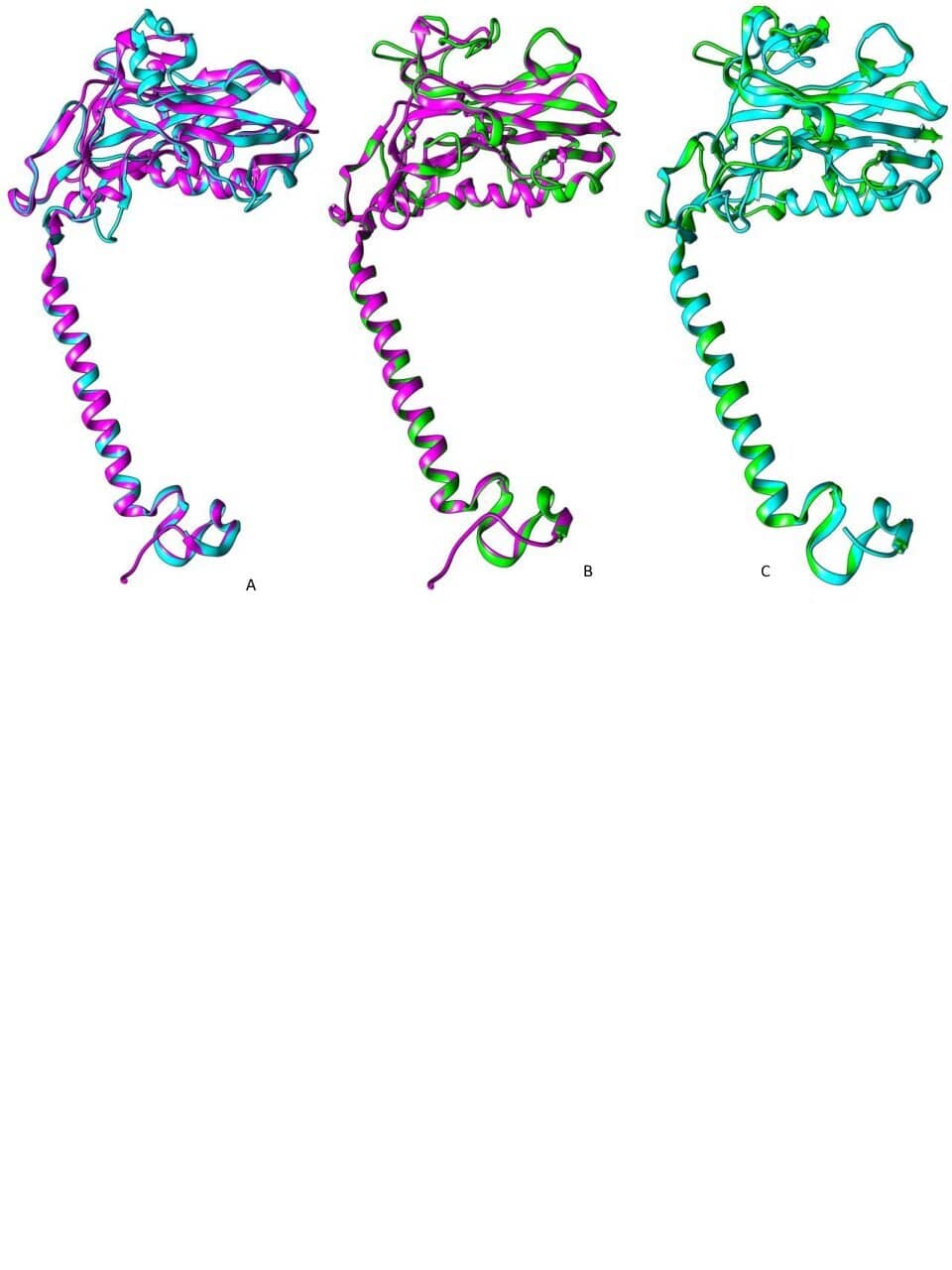

Supplement: Supplementary file 1 [file plants-10-00221-s001.zip › Supplementary figure 2_alignment-3D.jpg]
